# Tracer kinetic models as temporal constraints during brain tumor DCE‐MRI reconstruction

**DOI:** 10.1002/mp.13885

**Published:** 2019-11-19

**Authors:** Sajan Goud Lingala, Yi Guo, Yannick Bliesener, Yinghua Zhu, R. Marc Lebel, Meng Law, Krishna S. Nayak

**Affiliations:** ^1^ Roy J Carver Department of Biomedical Engineering University of Iowa Iowa City IA USA; ^2^ Snap Inc. San Francisco CA USA; ^3^ Ming Hsieh Department of Electrical and Computer Engineering University of Southern California Los Angeles CA USA; ^4^ Google Inc. Boston MA USA; ^5^ GE Healthcare Applied Sciences Laboratory Calgary Canada; ^6^ Department of Neuroscience Monash University Melbourne Australia

**Keywords:** DCE-MRI, kinetic model based reconstruction, sparse sampling

## Abstract

**Purpose:**

To apply tracer kinetic models as temporal constraints during reconstruction of under‐sampled brain tumor dynamic contrast enhanced (DCE) magnetic resonance imaging (MRI).

**Methods:**

A library of concentration vs time profiles is simulated for a range of physiological kinetic parameters. The library is reduced to a dictionary of temporal bases, where each profile is approximated by a sparse linear combination of the bases. Image reconstruction is formulated as estimation of concentration profiles and sparse model coefficients with a fixed sparsity level. Simulations are performed to evaluate modeling error, and error statistics in kinetic parameter estimation in presence of noise. Retrospective under‐sampling experiments are performed on a brain tumor DCE digital reference object (DRO), and 12 brain tumor *in‐vivo* 3T datasets. The performances of the proposed under‐sampled reconstruction scheme and an existing compressed sensing‐based temporal finite‐difference (tFD) under‐sampled reconstruction were compared against the fully sampled inverse Fourier Transform‐based reconstruction.

**Results:**

Simulations demonstrate that sparsity levels of 2 and 3 model the library profiles from the Patlak and extended Tofts‐Kety (ETK) models, respectively. Noise sensitivity analysis showed equivalent kinetic parameter estimation error statistics from noisy concentration profiles, and model approximated profiles. DRO‐based experiments showed good fidelity in recovery of kinetic maps from 20‐fold under‐sampled data. *In‐vivo* experiments demonstrated reduced bias and uncertainty in kinetic mapping with the proposed approach compared to tFD at under‐sampled reduction factors >= 20.

**Conclusions:**

Tracer kinetic models can be applied as temporal constraints during brain tumor DCE‐MRI reconstruction. The proposed under‐sampled scheme resulted in model parameter estimates less biased with respect to conventional fully sampled DCE MRI reconstructions and parameter estimation. The approach is flexible, can use nonlinear kinetic models, and does not require tuning of regularization parameters.

## 1 Introduction

Dynamic contrast enhanced‐magnetic resonance imaging (DCE‐MRI) is a powerful technique that provides a quantitative measure of vessel permeability and interstitial volumes. In the brain, it characterizes the blood brain barrier (BBB) leakiness, which has proven to be valuable in several applications.^1^ These include assessing conditions with large BBB breakdown such as gradation of brain tumors,^2^, ^3^ multiple sclerosis lesions,^4^, ^5^ and conditions with subtle and chronic BBB breakdown such as diabetes,^6^ and Alzheimer's disease.^7^ Outside the brain, DCE‐MRI has applications in cancer assessment and therapeutic monitoring in several body parts including breast,^8^, ^9^ prostate,^10^ and liver.^11^

DCE‐MRI involves a challenging trade‐off between the achievable spatial resolution, temporal resolution, and volume coverage. Acceleration strategies that exploit redundancies along the time dimension have shown significant potential to improve these trade‐offs. These include schemes such as view‐sharing,^12^, ^13^, ^14^ highly constrained back projection (HYPR),^15^ and compressed sensing.^16^, ^17^, ^18^, ^19^, ^20^, ^21^ Several sparsifying spatio‐temporal transforms have been proposed including spatio‐temporal wavelet transform, spatio‐temporal finite‐difference, temporal Fourier transform. A major challenge with these “off‐the‐shelf" object models is that the modeling assumptions do not fit the data, which limits the achievable acceleration rates. Data‐driven schemes that learn sparse representations from the data have been proposed,^22^, ^23^, ^24^, ^25^ and have shown to out perform off‐the shelf transforms. However, these are often associated with highly nonconvex optimization. Furthermore, image reconstruction with existing transforms involves tuning one or more regularization parameters, which poses challenges to the standardization of these methods.

In this manuscript we explore the use of physical tracer kinetic models for constrained reconstruction. This approach has been used extensively in dynamic positron emission tomography (PET) imaging,^26^, ^27^, ^28^, ^29^ and has recently been adapted in MRI for the applications of relaxometry,^30^, ^31^, ^32^, ^33^ perfusion,^34^, ^35^ permeability,^36^, ^37^ and diffusion imaging.^38^, ^39^ Broadly, these methods can be classified into methods based on direct reconstruction of parameters from under‐sampled data, or methods that use representations derived from parametric models as constraints in image reconstruction.

We propose a model‐constrained approach for DCE‐MRI reconstruction, where established contrast‐agent kinetic models used in post‐processing are employed as temporal constraints in reconstruction. From a specific kinetic model, and a physiological range of kinetic parameters, we construct a library of concentration vs. time profiles. Kinetic model‐specific temporal basis functions are derived from the library using the k‐singular value decomposition (k‐SVD) algorithm.^40^ Through noiseless and noise‐based simulations, we deduce a relation between the sparsity parameter in k‐SVD and the complexity level of the kinetic model. We design a constrained reconstruction method where the kinetic model‐based temporal bases are used to constrain the recovery of concentration vs time profiles from under‐sampled (k‐t) data. We utilize an iterative multiscale optimization algorithm for improved robustness to undesirable local minima solutions.

The proposed approach has similarities with recent work on direct reconstruction of kinetic parameters from under‐sampled DCE‐MRI data.^36^, ^37^ We use the same tracer kinetic model for reconstruction and post‐processing to exploit the redundancy in the DCE‐MRI pipeline. The major difference is in formulation of the optimization problem. Direct reconstruction involves estimation of kinetic parameters directly from under‐sampled data.^36^ When using the Patlak model, a Newton‐based solver is used. When using the ETK model^37^ a variable splitting strategy is used to iterate between sub‐problems of data consistency, concentration time profile estimation, and kinetic parameter estimation. One major challenge is that the kinetic parameter estimation is treated as a black box. For models like ETK, this fitting problem is nonlinear and is applied to concentration vs. time profiles containing noise and potential artifact at every iteration. Errors in kinetic parameter estimation propagate to the main reconstruction step. A second limitation is substantial compute times as kinetic model estimation is performed at every iteration. In contrast, the proposed approach decouples kinetic parameter estimation from the reconstruction of concentration profiles. This avoids calling the computationally expensive kinetic parameter estimation during reconstruction. The proposed optimization iterates between data consistency, concentration profile estimation, and k‐sparse projection of concentration profiles onto a set of temporal basis functions. Kinetic parameter estimation needs to be performed only once.

Since our formulation decouples reconstruction of concentration profiles from parameter estimation, it allows for flexibility to adapt to complex nonlinear kinetic models. Furthermore, since the sparsity parameter is fixed *a priori*, the proposed approach does not require any tuning of free parameters (e.g., regularization parameters). The flexibility allows for its potential utility in DCE‐MRI of most organs and disease conditions. In this work, we demonstrate effectiveness with both the Patlak and extended Tofts‐Kety (ETK) models, and demonstrate application to brain tumor assessment.

## 2 Materials and methods

### 2.A. Tracer kinetic model‐based temporal bases

**Figure 1:**
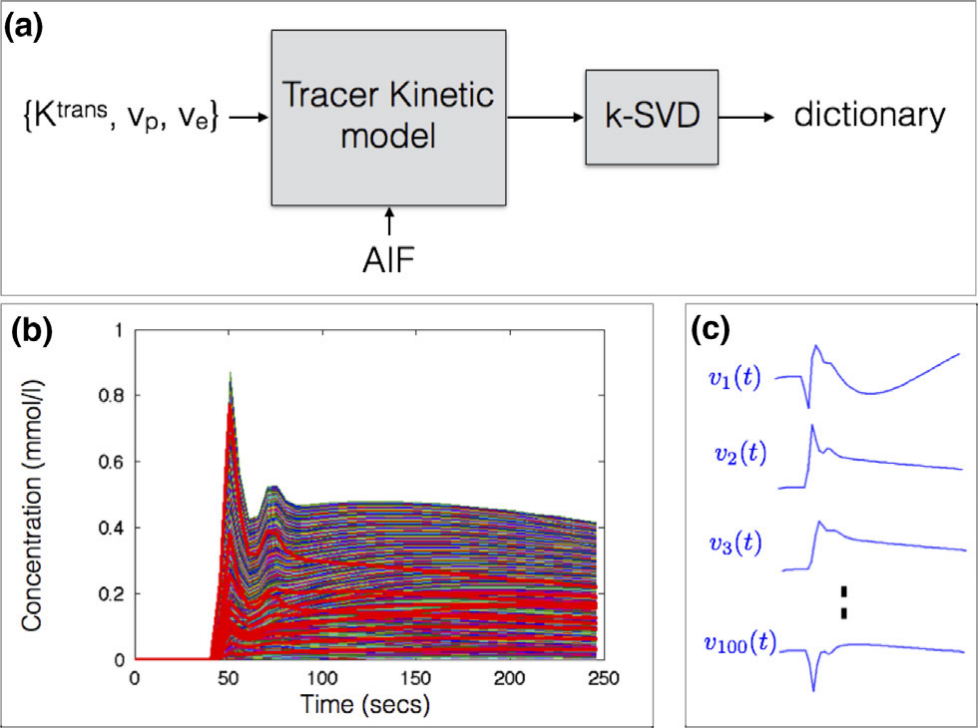
Construction of dictionary of temporal basis functions from a specified tracer kinetic model (a). Based on a physiological range of kinetic parameters and an arterial input function (AIF), a library of concentration vs time profiles is generated (b). A subset of the profiles in the library is highlighted in red. Using k‐SVD, the library is then reduced to a smaller set of temporal basis functions in a dictionary (c). The basis functions generated with the extended Tofts‐Kety model is shown in (c). The basis functions themselves are not representative of kinetic model profile, and hence can be nonpositive. Instead, the linear combination of them is designed to mimic any profile in (b). Approximate MATLAB computational times respectively for generating the library (~400 000 profiles) and learning the dictionary were 11.5 min and 3.5 h. [Color figure can be viewed at http://www.wileyonlinelibrary.com]

A library of concentration vs. time profiles $Z_{lxN}$ is simulated using a kinetic model, an arterial input function (AIF), and a physiologic range of kinetic parameters (Fig. 1). l denotes the number of profiles in the library; and N denotes the number of time instances. For the ETK model,^41^ we used the range: $K^{\mathrm{trans}}=0-0.8\min^{-1}$ in steps of $0.01\min^{-1}$, $v_{p}=0-60\%$ in steps of $1\%,v_{e}=0-100\%$ in steps of 1% to yield a library of size l×N=494100×50. Similarly, for the Patlak model,^42^ we used the range: $K^{\mathrm{trans}}=0-0.8\min^{-1}$ in steps of $0.01\min^{-1}$, $v_{p}=0-60\%$ in steps of 1% to yield a library size l×N=4941×50. We assume a hematocrit (hct) of 0.4, which is equivalent to the range of blood volume (v_b_) between 0% and 100% as v_b_ = v_p_/(1 ‒ Hct). N was chosen as 50 to match our *in‐vivo* DCE‐MRI acquisition settings (i.e., temporal resolution of 5 s and the total scan time of 250 s). This can however be adjusted based on the temporal resolution and scan time of the DCE‐MRI acquisition. A population‐based AIF was used.^43^ The settings of the Parker model that specifies the populationbased AIF were the same as described in Ref. [43]. The range of kinetic parameters was motivated by brain tumor DCE literature,^1^ which suggests 0‐0.34 min^‒1^ for K^trans^, 0%–60% for v_p_ assuming hematocrit of 0.4, and 0%–100% for v_e_. We expanded the K^trans^ range by ~2.5x, and used the full range for v_p_ and v_e_ to ensure conservative coverage of the kinetic parameter space. The k‐SVD dictionary‐learning algorithm^40^ is then used to reduce the large library to a smaller dictionary of temporal basis functions (denoted by $V_{r\text{xN}}$). k‐SVD represents any time profile in Z, for instance the *p*th row of Z, z*_p_*(*t*) as a sparse linear combination of basis functions $v_{i}\left(t\right)$ from V:

(1) $$\underset{1\times N}{\underbrace{z_{p}\left(t\right)}}\approx \underset{z_{p}^{q-sp}\left(t\right)}{\underbrace{u_{p}V_{r\times N}}}\;\text{such that,}\left||\underset{r\times 1}{\underbrace{u_{p}}}\right|{|}_{0}\leq q;$$

where r denotes the number of basis functions in V, and is chosen as r=100≪l. q is the sparsity parameter. $\left|\right|u_{p}{\left|\right|}_{0}$ denotes the l_0_ norm of the vector $u_{p}=\left\{u_{1},u_{2},\ldots ,u_{r}\right\}$. $z_{p}^{q-sp}(t$) denotes the q‐sparse projection of $z_{p}\left(t\right)$ onto V. k‐SVD jointly estimates the sparse coefficient matrix $U_{lxr}$ and the dictionary $V_{rxN}$ as:

(2) $$\left\{{\hat{U}}_{l\times r},{\hat{V}}_{r\times N}\right\}=\min_{\text{U,V}}\sum_{p=1}^{l}{\left||z_{p}\left(t\right)-u_{p}V\right||}_{2}^{2};\;\text{such that},\;\left||u_{p}\right|{|}_{0}\leq q;$$

where $u_{p}$ denotes the *p*th row of U.

### 2.B. Image Reconstruction

We pose the estimation of the concentration vs time profiles $C_{MxN}$ (M — number of pixels; N — number of time frames), and the sparse coefficient matrix $U_{Mxr}$ from under‐sampled k‐t space data (b) as:

(3) $$min_{C,U}\underset{\text{data consistency}}{\underbrace{\left||A\left(C\right)-b\right|{|}_{2}^{2}}};s.t.,\underset{\text{TK model constraint}}{\underbrace{C=\text{UV};\left||u_{p}\right|{|}_{0}\leq q;p=\left\{1,2,\ldots ,M\right\};}}$$

C contains the concentration v.s time profile $c\left(x,t\right)$ for every pixel $x\in \left(x,y\right)$ stacked row wise. $c\left(x,t\right)$ are constrained to be a q‐sparse linear combination of the kinetic model‐derived temporal bases in $V_{r\times N}$. The operator $A\left(C\right)=F_{u}\left(S_{m}\left(T\left(C\right)\right)\right)$ denotes the forward model which maps C to the measured multicoil (k,t) data. $F_{u}$ denotes the Fourier Transform operator on a specified (k‐t) under‐sampling pattern. $S_{m}$ contains the receiver coil sensitivity maps. To estimate the coil maps, the standard sum of squares method is applied on high SNR multicoil images obtained after gridding reconstruction of the time collapsed raw k‐t data; the coil maps are assumed to capture object phase. T is an operator that relates the concentration profile to the signal intensity profile $s\left(x,t\right)$ by the steady state spoiled gradient echo (SPGR) equation:

(4) $$\begin{array}{cc} s\left(x,t\right)= & T\left(c\left(x,t\right)\right)\\ = & \frac{M_{0}\left(x\right)\sin\alpha \left(1-e^{-TR\left[R_{1}\left(x,0\right)+c\left(x,t\right)r_{1}\right]}\right)}{1-\cos\alpha \left(e^{-TR\left[R_{1}\left(x,0\right)+c\left(x,t\right)r_{1}\right]}\right)}\\ & +\left[s\left(x,0\right)-\frac{M_{0}\left(x\right)\sin\alpha \left(1-e^{-TR\left[R_{1}\left(x,0\right)\right]}\right)}{1-\cos\alpha \left(e^{-TR\left[R_{1}\left(x,0\right)\right]}\right)}\right]; \end{array}$$

where $r_{1}$ is the contrast agent relaxivity, TR is the repetition time, α is the flip angle, $R_{1}\left(x,0\right)$ and $M_{0}\left(x\right)$ are respectively the pre‐contrast $R_{1}$(reciprocal of $T_{1}$) and the equilibrium longitudinal magnetization. $s\left(x,0\right)$ is the pre‐contrast first frame, which is fully sampled. The bracketed term in the second row of Eq. (5) resolves differences between the pre‐contrast signal $s\left(x,0\right)$ and the predicted pre‐contrast signal based on the baseline $R_{1}\left(x,0\right)$ and $M_{0}\left(x\right)$ maps (from a separate $T_{1}$ mapping acquisition). Similarly, the operation of mapping concentration profile from the signal intensity profile can be expressed as^44^:

(5) $$c\left(x,t\right)=T^{-1}\left(s\left(x,t\right)\right)=\frac{-\frac{1}{\mathrm{TR}}\ln\left[\frac{1-\left(\frac{s\left(x,t\right)-s\left(x,0\right)}{s\left(x,0\right)\sin\alpha }+\frac{1-e^{-TR\left[R_{1}\left(x,0\right)\right]}}{1-\cos\alpha \left(e^{-TR\left[R_{1}\left(x,0\right)\right]}\right)}\right)}{1-\cos\alpha \left(\frac{s\left(x,t\right)-s\left(x,0\right)}{s\left(x,0\right)\sin\alpha }+\frac{1-e^{-TR\left[R_{1}\left(x,0\right)\right]}}{1-\cos\alpha \left(e^{-TR\left[R_{1}\left(x,0\right)\right]}\right)}\right)}\right]-R_{1}\left(x,0\right)}{ℜ1};$$

We solve (4) by alternately (a) updating **U** using orthogonal matching pursuit (OMP) sparse projection,^40^, ^45^ and (b) updating **C** by enforcing consistency with acquired data. To be robust to spurious local minima, we use an iterative multiscale minimization approach, where we solve the problem at a coarser spatial resolution during the initial iterations and as the iterations proceed, we gradually update the resolution to its full resolution. This is achieved by multiplication of spatial Fourier Transform of $s\left(x,t\right)$ by a two‐dimensional Gaussian filter ($G\left(k_{\sigma }\right)$) specified by filter width $k_{\sigma }$; where $k_{\sigma }$ is initialized to 0.1% of k_max_, and gradually updated to 100 percent of k_max_, where k_max_ specifies the extent of k‐space coverage. This heuristic strategy is used in several nonconvex problems such as in image registration,^46^ and recently in MR‐fingerprinting.^47^, ^48^ Starting with an initial guess obtained from $C_{\text{init}}=A^{H}\left(b\right)$, we iterate until a stopping criterion of ${\left|\frac{\left||C_{i}-C_{i-10}\right||}{\left||C_{i}\right||}\right|}_{2}^{2}<\varepsilon =0.01$ or until the maximum number of iterations of 150 are achieved. After reconstructing $\hat{C}$, we estimate the kinetic parameters by fitting the estimated concentration profiles to the kinetic model using the open source Rocketship^49^ package. The pseudo code of the algorithm is shown below. The code and examples of the algorithm are publicly available at the following URL: https://github.com/sajanglingala/DCE_dictionary_recon/.

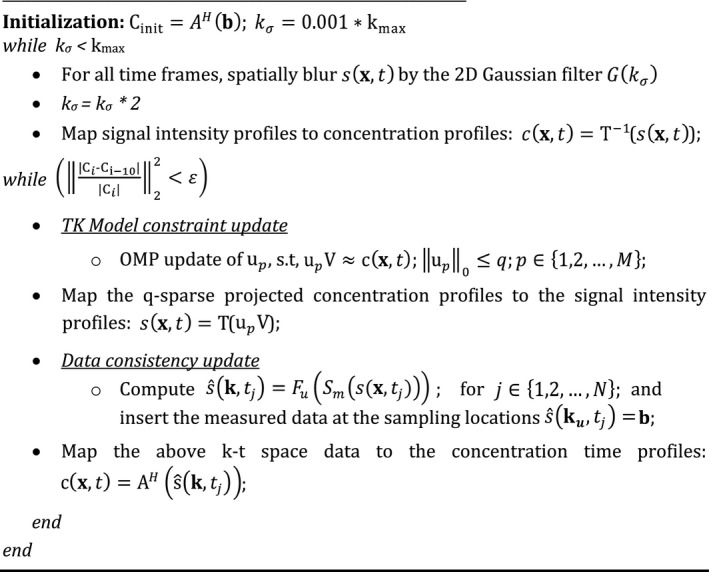

### 2.C. Simulations

The sparsity parameter q in Eq. (2) is determined based on simulation studies with the Patlak and the ETK models. Noiseless simulations are performed and the mean approximation error $\mu_{\mathrm{err}}=\frac{1}{l}\sum_{p=1}^{l}\left||z_{p}\left(t\right)-z_{p}^{q-sp}\left(t\right)\right|{|}_{2}^{2}$, and maximum approximation error: $max_{\mathrm{err}}=max_{p=1}^{l}\left||z_{p}\left(t\right)-z_{p}^{q-sp}\left(t\right)\right|{|}_{2}^{2}$ are computed for different values of q.

Noise‐based simulations were performed for broad ranges of kinetic parameter values to (a) determine any systematic bias and uncertainty in the kinetic parameter space that may be induced by sparsity‐based modeling of the concentration time profiles, and (b) to deduce the correspondence between the sparsity level (q) and the kinetic model.

Noisy concentration profiles were obtained as:

(6) $$z_{p}^{n}\left(t\right)=z_{p}\left(t\right)+n\left(t\right);p=\left\{1,\ldots ,l\right\};$$

where $n\left(t\right)$ denotes i.i.d. white Gaussian noise with zero mean and 0.005 standard deviation, which was chosen to match the typical signal‐to‐noise ratio (SNR) from *in vivo* brain DCE‐MRI data acquired at our institution on a 3T commercial system with an eight‐channel head array coil. Noise in the concentration time profiles was assumed to be additive i.i.d. Gaussian. Concentration time profiles are real valued (negative values can occur in the presence of noise), and have a one‐to‐one mapping with real‐valued signal intensity time profiles in the forward model. Monte‐Carlo simulations with 500 realizations of $n\left(t\right)$ were performed to evaluate the bias and uncertainty in estimating kinetic parameters from (a) the noisy profiles $z_{p}^{n}\left(t\right)$, and (b) the q — sparse projections of $z_{p}^{n}\left(t\right)$ on V: $z_{p}^{n,q-sp}\left(t\right)$.

We performed covariant error analysis for two parameters (K^trans^, v_p_) with the Patlak and the ETK model over a broad range of kinetic parameters. With both the models, we evaluated the bias and uncertainty in estimating K^trans^ and v_p_ before and after q‐sparse projections. With the ETK model, for simplicity, we focus only on analysis in a two dimensional space with a fixed v_e_ = 0.6. The open‐source Rocketship package^49^ was used for kinetic parameter estimation.

### 2.D. Evaluation with a digital reference object

An anatomically realistic brain tumor DCE‐MRI digital reference object (DRO) was generated based on the method and data described in Ref. [50]. Briefly, the population‐based AIF with the Parker model, known kinetic parameters, the ETK model, and the steady state spoiled gradient signal equation was used to generate the dynamic images. We then multiplied by coil sensitivities, took the Fourier Transform, and added realistic complex Gaussian noise to each channel. Coil maps, noise covariance matrix, and the signal to noise (SNR) level were obtained from *in‐vivo* data acquired at 3T. Comparisons were performed at a SNR = 30 to mimic measurements at 3T.

This phantom data was retrospectively under‐sampled using a randomized golden‐angle Cartesian (GOCART) sampling pattern,^51^ and evaluations in fidelity of the kinetic parameters were performed at under‐sampling factor of R = 20. GOCART^51^ is originally a three‐dimensional (3D) golden angle Cartesian sampling scheme, with random sampling of the ky‐kz phase encode locations along each Cartesian radial spoke. In this study, we perform retrospective under‐sampling in the kx‐ky plane in a representative slice. This strategy was chosen to simulate k_y_‐k_z_ under‐sampling in prospective acquisitions.

Reconstruction was performed using the fully sampled direct inverse Fourier Transform‐based approach (considered as reference), and the proposed constrained reconstruction approach with full‐sampling (R = 1), and under‐sampling (R = 20). Kinetic modeling was performed both using the ETK model, and a simpler Patlak model. The latter was used to analyze any errors due to model mismatch. The proposed constrained reconstruction was implemented with temporal dictionaries derived from the kinetic model (Patlak or ETK) that corresponded to the same kinetic model used for subsequent parameter estimation.

### 2.E. Evaluation with *in‐vivo* data

We reviewed 110 fully sampled DCE‐MRI raw datasets from patients with known or suspected brain tumor, receiving a routine brain MRI with contrast on a clinical 3T scanner (HDxt, GE Healthcare, Waukesha, WI). The data acquisition was based on a 3D Cartesian SPGR sequence with field of view (FOV): 22 × 22 × 4.2 cm^3^; spatial resolution: 0.9 × 1.3 × 7.0 mm^3^; temporal resolution: 5 s; 50 time frames; and eight receiver coils; flip angle of 15°, TE/TR = 1.3 ms/6 ms. Driven equilibrium Single Pulse Observation of T1 (DESPOT1) was performed before the DCE sequence, where three images with flip angles of 2°, 5°, 10° were acquired to estimate T1 and M0 maps before the contrast arrival. Gadobenate dimeglumine (Multihance, Bracco) (0.05 mmol/kg) was administered into an upper extremity vein using a power injector (ACIST EmpowerMR Injector, Bracco), at a rate of 3 ml/s, followed by a 20 ml saline flush.

**Figure 2:**
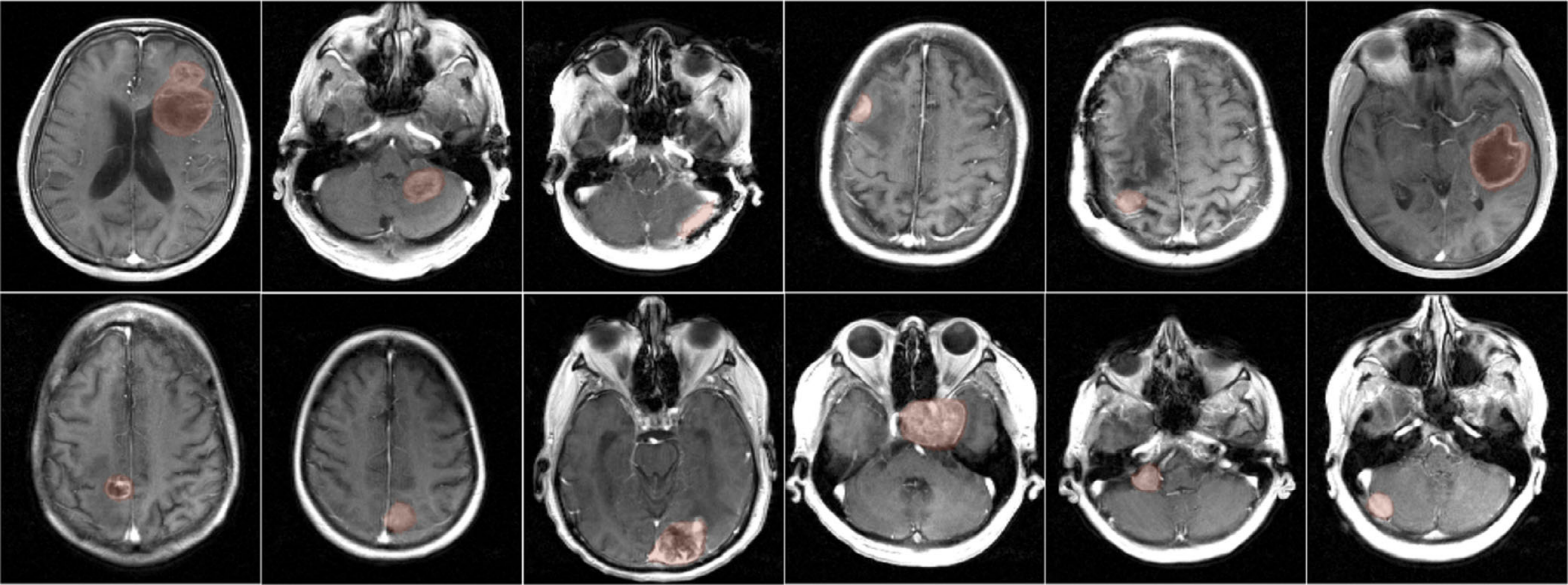
Post‐contrast images of 12 brain tumor cases with different brain tumor characteristics (shape, size, heterogeneity). All cases had enhancing tumors of at least 1 cm as determined by standard bi‐directional assessment.^52^ Fully sampled raw multicoil (k‐t) space data from these patients were used as reference in retrospective under sampling studies. Tofts‐Kety parameter estimation was performed in the tumor regions of interests as marked by the red shaded regions. [Color figure can be viewed at http://www.wileyonlinelibrary.com]

Of these 110 cases, we identified a cohort of 12 cases, which had different brain tumor characteristics (shape, size, heterogeneity), and also had enhancing tumors of atleast 1 cm (as determined by standard bi‐directional assessment).^52^ The demographics of these patients are shown in Table 1, and the post‐contrast images (last spatial frame from the DCE‐scans) are shown in Fig. 2. The protocol was approved by our institutional review board (IRB).

**Table 1:** Patient demographic information and diagnosis of the brain tumor cases used in this study.

Case no.	Age/sex	Diagnosis
1	74/M	Glioblastoma
2	60/M	Metastatic Melanoma
3	44/F	Meningioma
4	79/F	Metastatic melanoma
5	63/M	Meningioma
6	68/M	Glioblastoma
7	73/M	Metastatic melanoma
8	38/F	Meningioma
9	67/M	Renal Cell Carcinoma
10	71/M	Pituitary adenoma
11	73/F	Meningioma
12	54/F	Meningioma

(k‐t) under‐sampling was performed retrospectively on fully sampled raw data using the GOCART (randomized golden angle Cartesian) sampling trajectory^51^ at acceleration factors R = 20 and R = 40. Image reconstruction was performed with the proposed dictionary based approach, an existing compressed sensing approach that uses a temporal finite difference (tFD) sparsity constraint,^19^ and compared against the reference fully sampled inverse Fourier Transform based approach. ETK derived bases with a fixed sparsity level of q = 3 was used in the proposed approach. The ETK model was chosen as it accounts for backflux of contrast from the extravascular space to the plasma, which in turn improves the accuracy of $K^{\mathrm{trans}}$ estimation, and has shown to be applicable to brain tumor data.^53^ All the patient datasets were acquired with fixed injection timing, however timing delays between 5 and 10 s (1–2 frames) existed amongst different patients. As described earlier, a population based AIF with a fixed delay was used to generate the library. Patient‐specific AIF delays were estimated as described by Lebel et al.^54^ Briefly, the k‐space origin was frequently sampled, and plotted as a function of time. The region of maximum slope was regressed to the baseline to determine the bolus arrival time. Either padding zeroes initially to the acquired data or omitting the last time frames corrected for any delay mismatch to the library. The tFD‐based formulation is convex and is guaranteed to achieve the global minimum. Therefore the multiscale optimization heuristic was not applied during tFD optimization. We used the alternating direction method of multipliers (ADMM) algorithm where the stopping criterion was if the rate of change between reconstructions at successive iterations fell below 10^−5^ percent. The regularization parameter in tFD constrained reconstruction was tuned to provide the smallest normalized root mean squared image reconstruction error (nRMSE) in tumor ROIs with respect to the reference fully sampled datasets. All reconstructions were implemented in MATLAB (The MathWorks, Inc., Natick, MA) and executed on an Intel core i7 3.5 GHz machine with 32 GB memory.

The convergence of the proposed multiscale iterative optimization was evaluated empirically. Reconstruction estimates with different initializations of the concentration profiles were compared: zero filled reconstruction ($C_{\text{init}}=A^{H}\left(b\right))$; low spatial resolution estimate obtained from the center 3x3 window of the k‐space data in every time frame ($C_{\text{init}}=C_{\text{low}.\text{res}})$; and from the reference fully sampled data ($C_{\text{init}}=C_{\text{ref}})$;

After image reconstruction, the ETK model was used to estimate the kinetic parameters with a population based AIF.^43^ Bland‐Altman analysis was performed to evaluate systematic bias and uncertainty of the reconstructed ${\hat{K}}^{\mathrm{trans}}$ and ${\hat{v}}_{p}$ maps (from the proposed and tFD approaches) with respect to the reference fully sampled $K_{\mathrm{ref}}^{\mathrm{trans}}$ and $v_{p,ref}$. Comparisons using $v_{e}$ maps were not considered, as its estimation is associated with high uncertainty with the ETK model.^49^

To evaluate error in the kinetic maps on using the ETK model to constrain the reconstruction of time intensity curves, we analyzed the kinetic maps from the proposed reconstruction against conventional direct inverse Fourier Transform reconstruction on fully sampled data (R = 1).

On one of the *in‐vivo* datasets where the Patlak model produced less than one percent modeling error with the population based AIF, the proposed reconstruction scheme implemented with the Patlak dictionary was also compared against the direct Patlak parameter estimation method.^36^ The direct estimation approach was implemented based on open source code (https://github.com/usc-mrel/DCE_direct_recon). The reconstruction quality in the resulting K^trans^ and v_p_ maps from under‐sampled data (at R = 10–30) were compared against the maps obtained by conventional fully sampled (R = 1) inverse Fourier Transform reconstructions.

## 3 Results

### 3.A. Simulations

**Figure 3:**
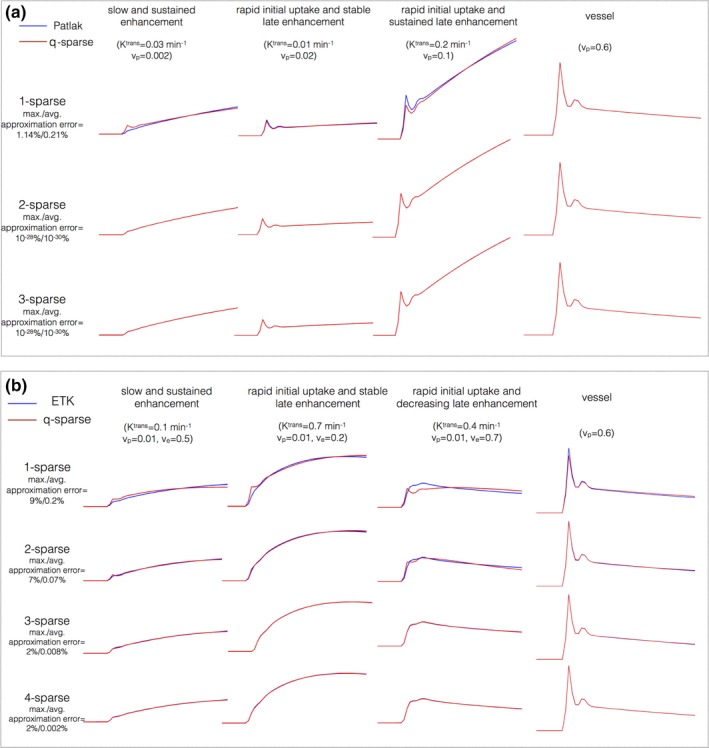
Kinetic model generated concentration vs time profiles and their representation using k‐SVD derived temporal bases. (a) and (b) respectively show representative profiles depicting different tumor enhancement dynamics from the Patlak, and extended Tofts‐Kety models. The maximum and average approximation errors are evaluated over the physiological range of kinetic parameters. A model sparsity choice of q = 2 was determined to be adequate for the Patlak model $max_{\mathrm{err}}/\mu_{\mathrm{err}}=10^{-28}\%/10^{-30}\%)$. Similarly, q = 3 was adequate for the ETK model $max_{\mathrm{err}}/\mu_{\mathrm{err}}=2\%/0.008\%)$. [Color figure can be viewed at http://www.wileyonlinelibrary.com]

Figure 3 shows the maximum and average approximation errors ($max_{\mathrm{err}},\;\text{and}\;\mu_{\mathrm{err}}$)between the concentration vs time profiles in the library, and the profiles obtained from q‐sparse projections onto V at different sparsity levels (q). q‐sparse projections of the curves generated from the Patlak and the ETK models are respectively shown in Figs. 3(a), and 3(b). These curves are chosen to represent different types of tumor enhancement dynamics.^55^ With q = 1, we observe considerable bias in approximating the kinetic model generated curves with both the Patlak and the ETK models. However, for the Patlak model, a choice of q ≥ 2 provided excellent agreement with the profiles in the library ($max_{\mathrm{err}}/\mu_{\mathrm{err}}=10^{-28}\%/10^{-30}\%)$. Similarly, for the ETK model, a choice of q ≥ 3 approximated the profiles in the library with ($max_{\mathrm{err}}/\mu_{\mathrm{err}}=2\%/0.008\%)$.

**Figure 4:**
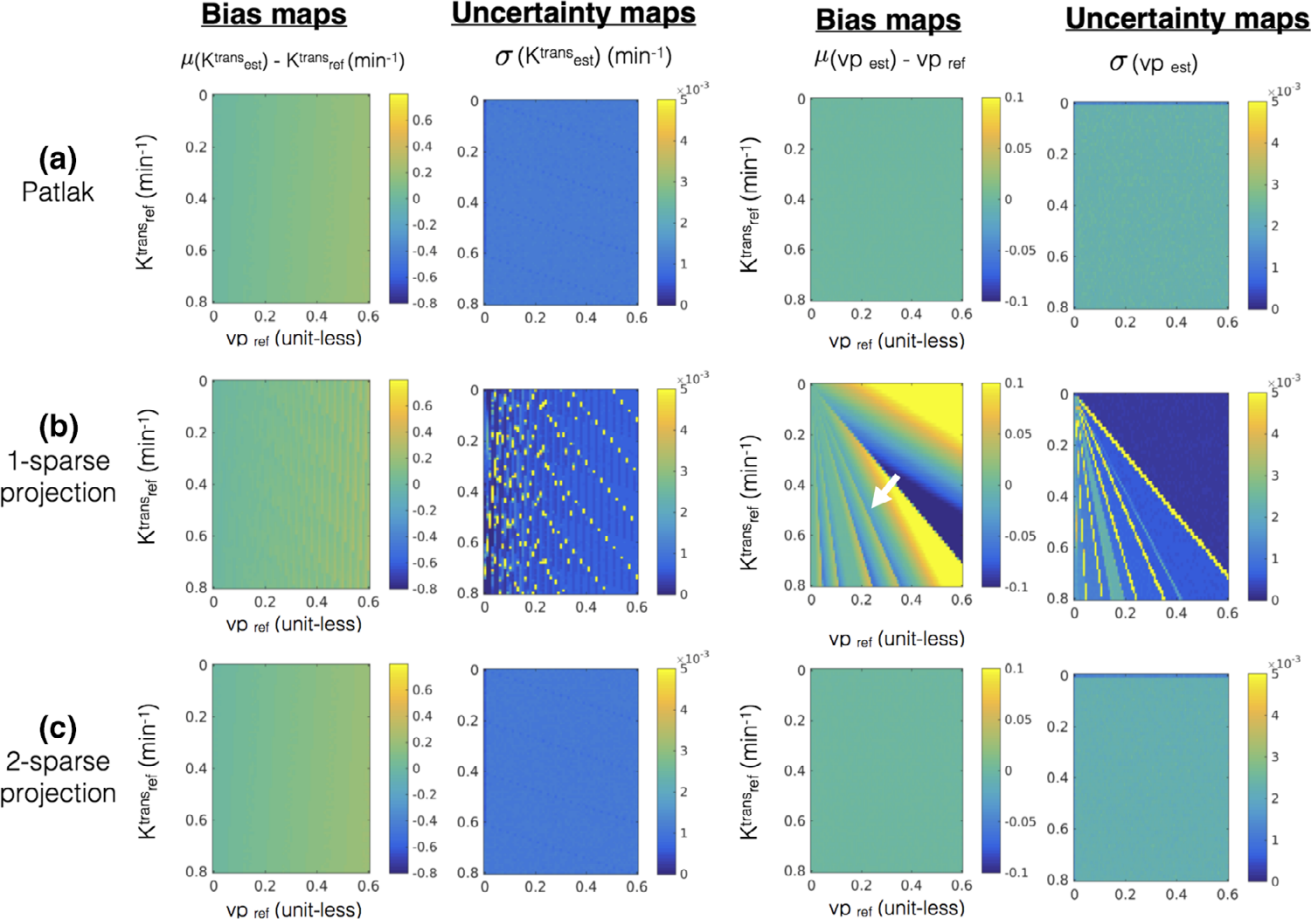
Error statistics (bias and uncertainty) in estimating kinetic parameters in the presence of noise with the Patlak model. The first row in (a) shows the bias and uncertainty in estimating kinetic parameters from the noisy concentration vs time profiles and is considered as reference. Rows (b) and (c) show the bias and uncertainty in kinetic parameter estimation after q‐sparse projection of the noisy profiles with different values of q, and is evaluated against the reference. It can be seen from (b) that q = 1 demonstrates considerable bias (e.g., see the white arrow in bias maps while estimating v_p_). However, when q = 2, the bias and uncertainty maps are equivalent to the reference, which motivated the choice of q = 2 for the Patlak model. [Color figure can be viewed at http://www.wileyonlinelibrary.com]

**Figure 5:**
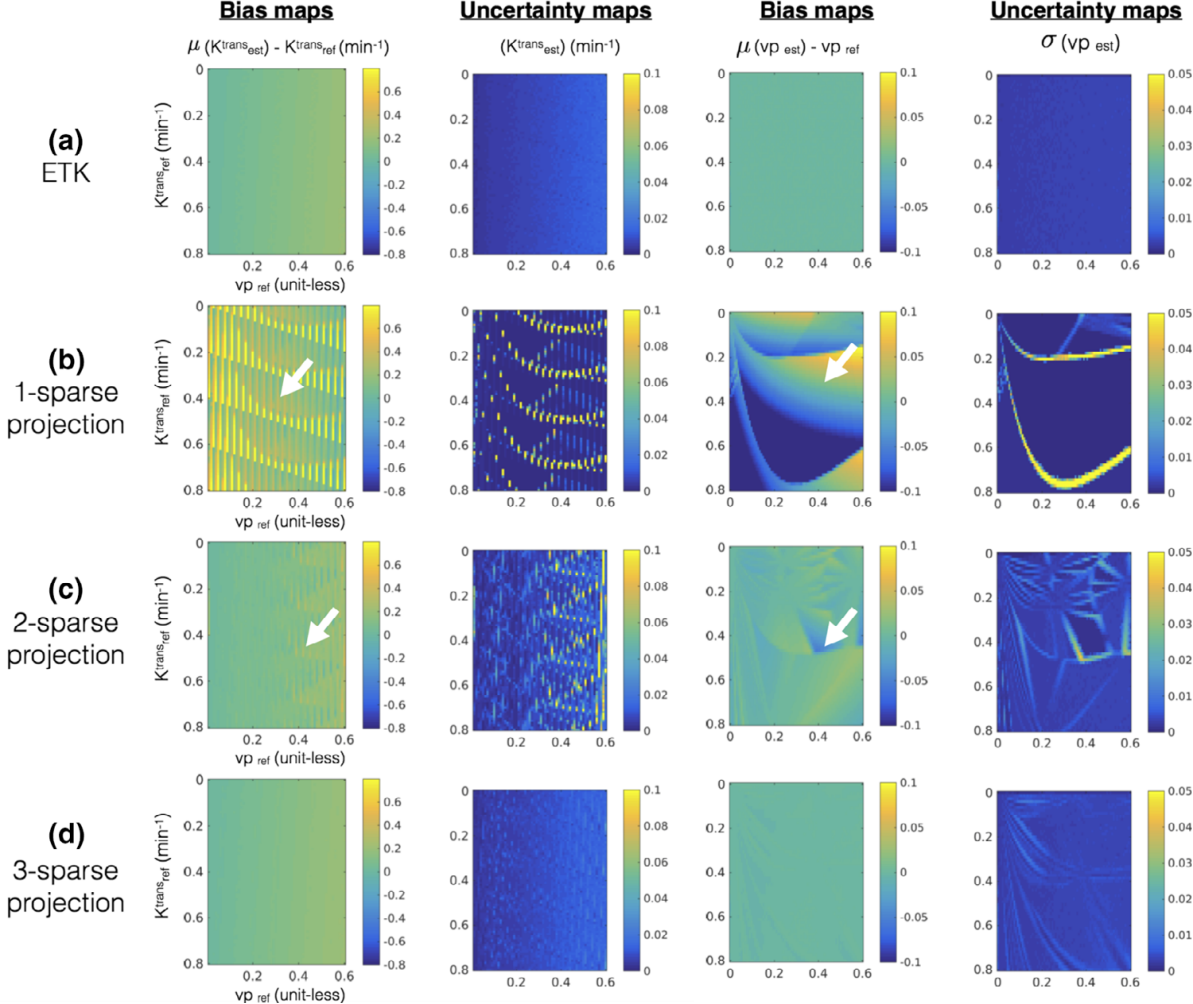
Error statistics (bias and uncertainty) in estimating kinetic parameters in presence of noise with the extended Tofts‐Kety model. The first row in (a) shows the bias and uncertainty in estimating kinetic parameters from the noisy concentration vs time profiles and is considered as reference. Rows (b–d) show the bias and uncertainty in kinetic parameter estimation after q‐sparse projection of the noisy profiles with different values of q and is evaluated against the reference. It can be seen from (b) and (c) that q = 1, and q = 2 demonstrates considerable bias and uncertainty (also see white arrows in (b) and (c)). However in (d), when q = 3, the bias and uncertainty maps are similar to the reference in (a) over a broad range of the parameter space. This motivated our choice of q = 3 for the ETK model. [Color figure can be viewed at http://www.wileyonlinelibrary.com]

Figure 4 and 5 demonstrates the bias and uncertainty in estimating kinetic parameters in presence of noise. Over a broad range of kinetic parameters, we observe that estimating the kinetic parameters from noisy profiles and the q‐sparse projected profiles are equivalent when q ≥ 2 (for the Patlak model), and q ≥ 3 (for the ETK model). Based on these simulations, we fixed q = 2 for the Patlak model, and q = 3 for the ETK model.

**Figure 6:**
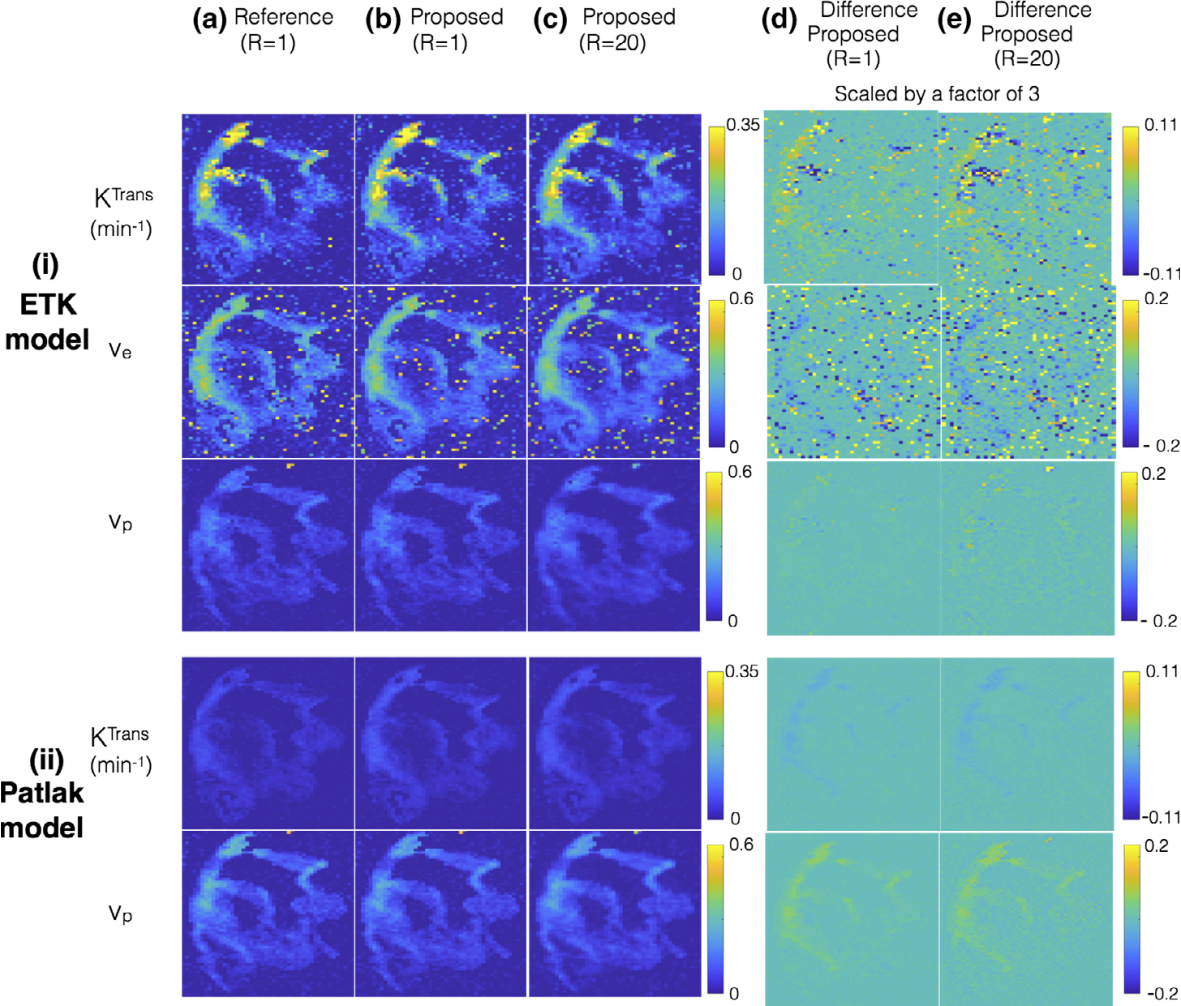
Evaluations using a brain tumor digital reference object (DRO). The DRO is constructed based on the extended Tofts‐Kety (ETK) model. (i) and (ii) shows the kinetic parameters after respectively applying the ETK and the Patlak model on the concentration time profiles obtained from (a) the reference fully sampled inverse Fourier Transform reconstruction (R = 1); (b) the proposed reconstruction applied on fully sampled data (R = 1); and (c) the proposed reconstruction applied on under‐sampled data (R = 20). The proposed reconstruction employs the ETK‐based dictionary in (i), and the Patlak‐based dictionary in (ii). From (i) and (ii), it is seen that when a simpler Patlak model is used in post‐processing, the resulting K^trans^ is under estimated in comparison to the ETK‐based K^trans^ estimates (about a factor of 10‐fold). This under estimation is observed in all the reconstructions (a–c) suggesting kinetic model selection error is independent of the type of the reconstruction scheme. From (a–c), it can be seen that proposed reconstruction shows good quality in the kinetic parameter estimates at R = 1 and R = 20, which is also highlighted in the difference maps scaled by a factor of 3 in (d–e). [Color figure can be viewed at http://www.wileyonlinelibrary.com]

Figure 6 shows evaluations on the brain tumor DRO which is constructed based on the ETK model. When the simpler Patlak model is applied on the reconstructed concentration time profiles, an under‐estimation in K^trans^ (a factor of about 10 fold) and over estimation in v_p_ (a factor of about 2.5 fold) is observed. This bias is observed in both the reference fully sampled inverse Fourier Transform reconstruction, and the proposed reconstruction suggesting that model selection error is independent of the choice of the reconstruction [see Fig. 6(b)].

When evaluated against the kinetic parameter estimates from reference reconstructions, the proposed reconstruction showed robust quality maps from 20 fold under‐sampled data [Figs. 6(a) and 6(b)]. This is also depicted in the difference maps where the reconstruction error lies at the level of background noise.

### 3.B. Evaluation with *in‐vivo* data

**Figure 7:**
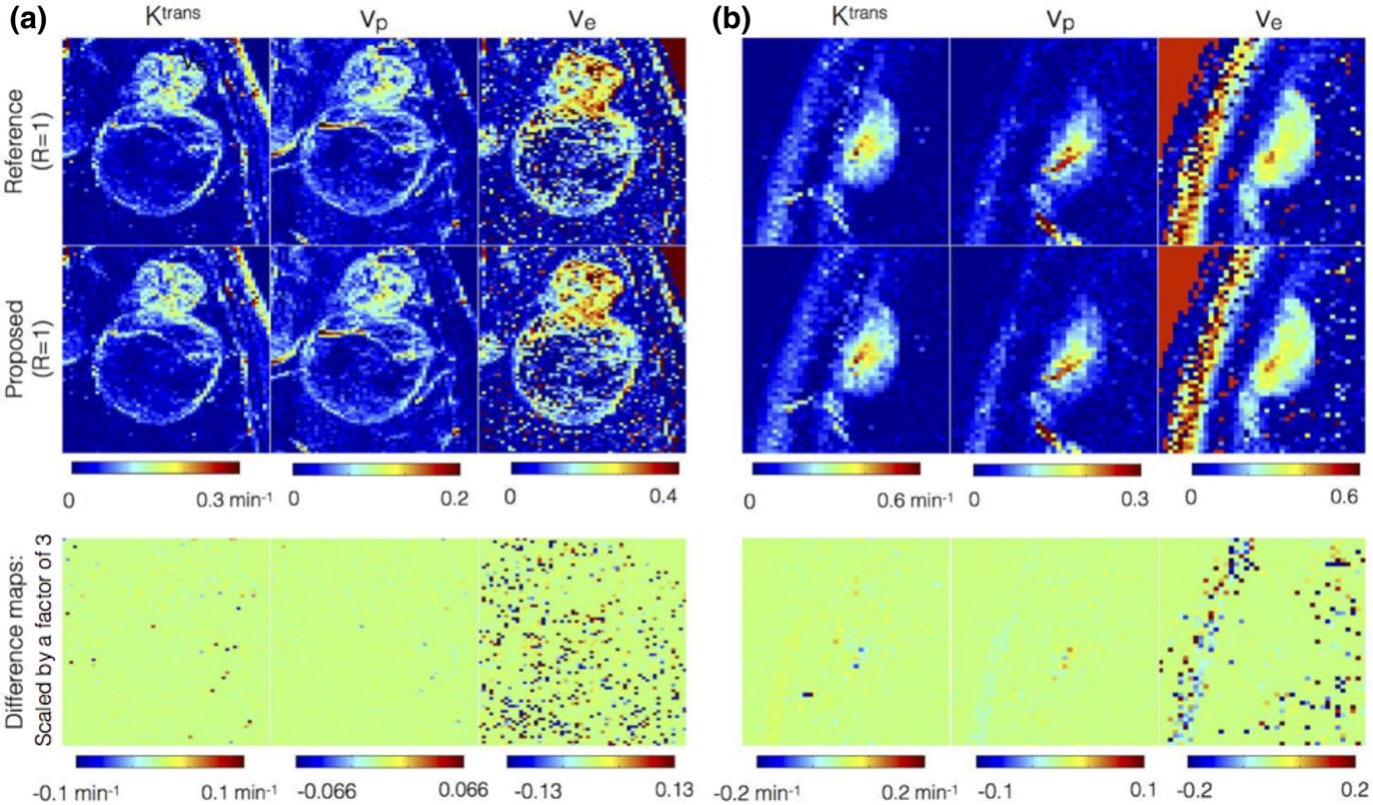
Comparison of kinetic parameters obtained from concentration time profiles from reference fully sampled data (first row) against the profiles after 3‐sparse projections onto the extended Tofts‐Kety (ETK) dictionary (second row). The third row denotes the difference map that highlights the kinetic modeling errors. The difference map is scaled up by a factor of 3 for better visualization. Two representative brain tumor datasets that have different spatial characteristics are shown: (a) glioblastoma with thin rim, necrotic core, connected to a solid tumor; (b) meningioma with homogenous spatial tumor characteristics. The kinetic maps in the first two rows are observed to be qualitatively equivalent suggesting 3‐sparse projection mimics ETK modeling. Compared to the K^trans^, and v_p_ maps, the uncertainty in v_e_ is large attributed to the short scan time duration of 5 min. [Color figure can be viewed at http://www.wileyonlinelibrary.com]

Figure 7 shows the kinetic maps from the proposed reconstruction against conventional direct inverse Fourier Transform reconstruction on fully sampled data (R = 1). Two representative brain tumor datasets that have different spatial characteristics are shown: (a) glioblastoma with thin rim, necrotic core, connected to a solid tumor; (b) meningioma with homogenous spatial tumor characteristics. As depicted in the kinetic maps, the maps obtained from the two approaches are qualitatively equivalent, with the error being in the background noisy regions. This is also highlighted in the difference maps. For both the reconstructions, the ve maps are noisy due to the increased uncertainity in estimating ve from short scan times (5 min in this study).^56^

**Figure 8:**
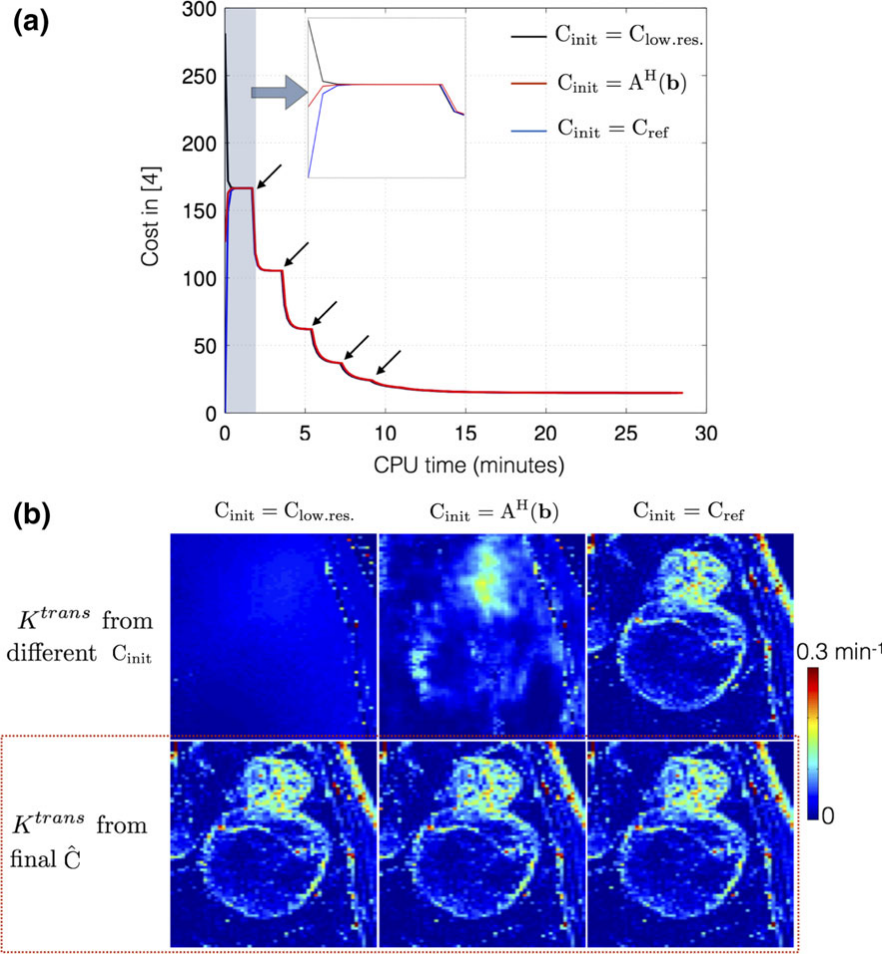
Convergence of cost function in Eq. (3): (a) shows the evolution of cost with different initializations of the concentration time profiles C). (b) demonstrates the K^trans^ estimated from the initial guesses (top row), and from the final estimated concentration time profiles (bottom row). Due to the iterative multiscale optimization, the algorithm ensures cycling through problems of increasing complexity. The black arrows in (a) indicate the instances at which the scale (spatial resolution) is incremented. It can be seen in (a) that the cost converges to the same minima irrespective of the initializations. The final estimated K^trans^ in (b) from the different initializations are identical (see red dotted box). [Color figure can be viewed at http://www.wileyonlinelibrary.com]

Figure 8 shows the evolution of the objective function in Eq. (3) as a function of CPU reconstruction time with different initializations of concentration time profiles (a) from low‐resolution dynamic images $C_{\text{low}.\text{res}.}$; (b) from zero‐filled dynamic images $C=A^{H}\left(b\right)$; (c) from reference dynamic images $C_{\text{ref}}$. The multiscale optimization gradually updates the complexity of the problem. Due to spatial low‐pass filtering, the under‐sampling artifacts in initial iterations are considerably reduced making the problem well‐posed. $\hat{C}$ is updated gradually with increasing resolution, as a result of which a monotonic convergence is observed. We empirically found this approach to be robust to local minima; the final solutions were identical with different initializations.

**Figure 9:**
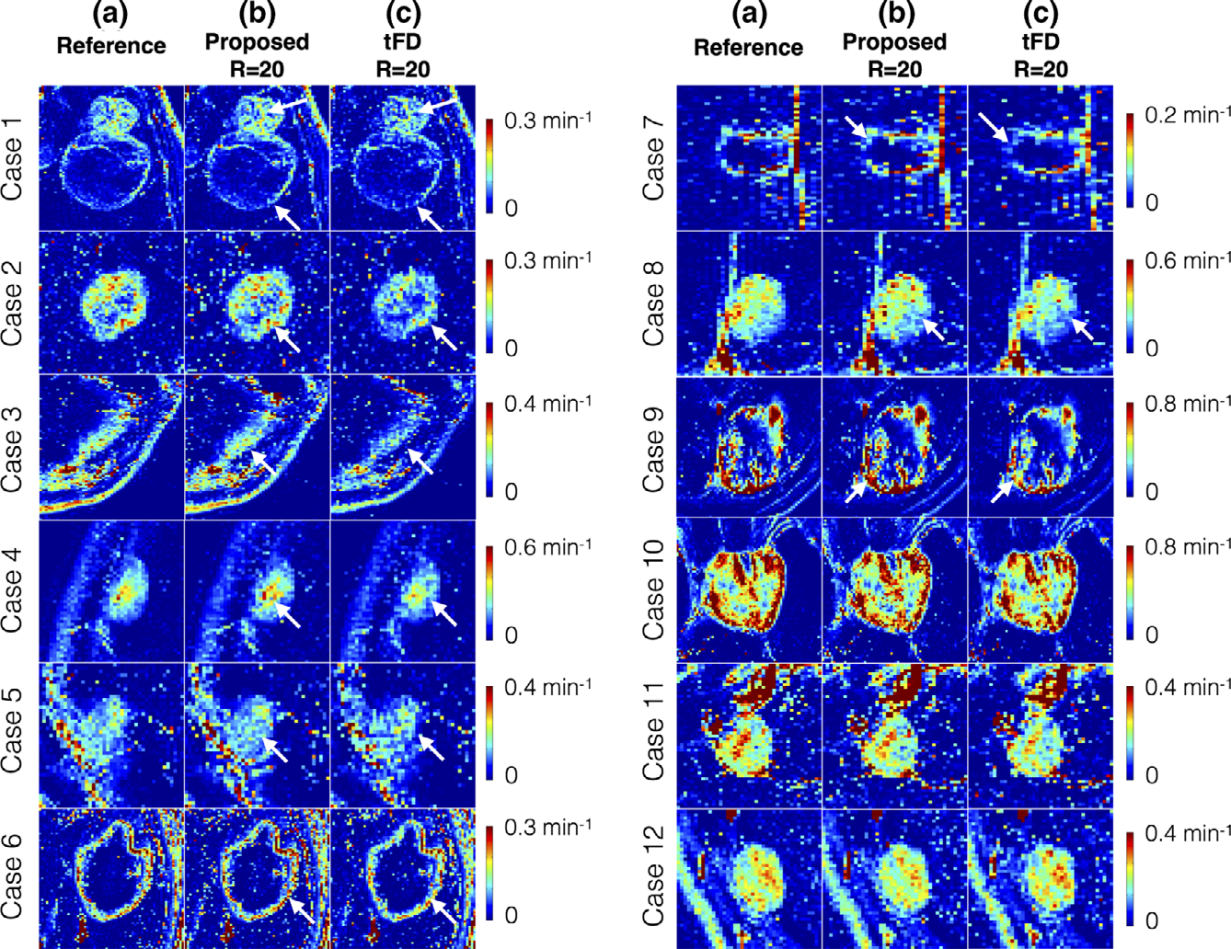
Evaluation of K^trans^ maps derived from the proposed dictionary‐based and tFD reconstructions (R = 20) against the reference K^trans^ maps (R = 1). The 12 cases are sorted based on decreasing difference between the proposed and tFD methods. The tFD reconstructions demonstrated under‐estimation of K^trans^ (visually evident in cases 1 to 9, see arrows). tFD also relied on tuning of a regularization parameter. In contrast, the proposed parameter‐free model‐based reconstruction provided K^trans^ estimates closer to that of the reference, and has improved fidelity in preserving spatial characteristics of the tumors (e.g., thin boundaries of the tumor, see arrows in cases 1–5). [Color figure can be viewed at http://www.wileyonlinelibrary.com]

Figure 9 shows retrospective under‐sampling comparisons of K^trans^ at R = 20. tFD reconstructions resulted in considerable under estimation of K^trans^ in 9 of the 12 cases, while the proposed method was found to be robust to this bias. tFD also relied on adjusting the regularization parameter. In contrast, the proposed parameter free reconstruction provided K^trans^ estimates closer to that of the reference. It also provided superior fidelity in maintaining spatial characteristics of the tumors in all cases (e.g., depiction of thin tumor boundaries in cases 1 to 5).

**Figure 10:**
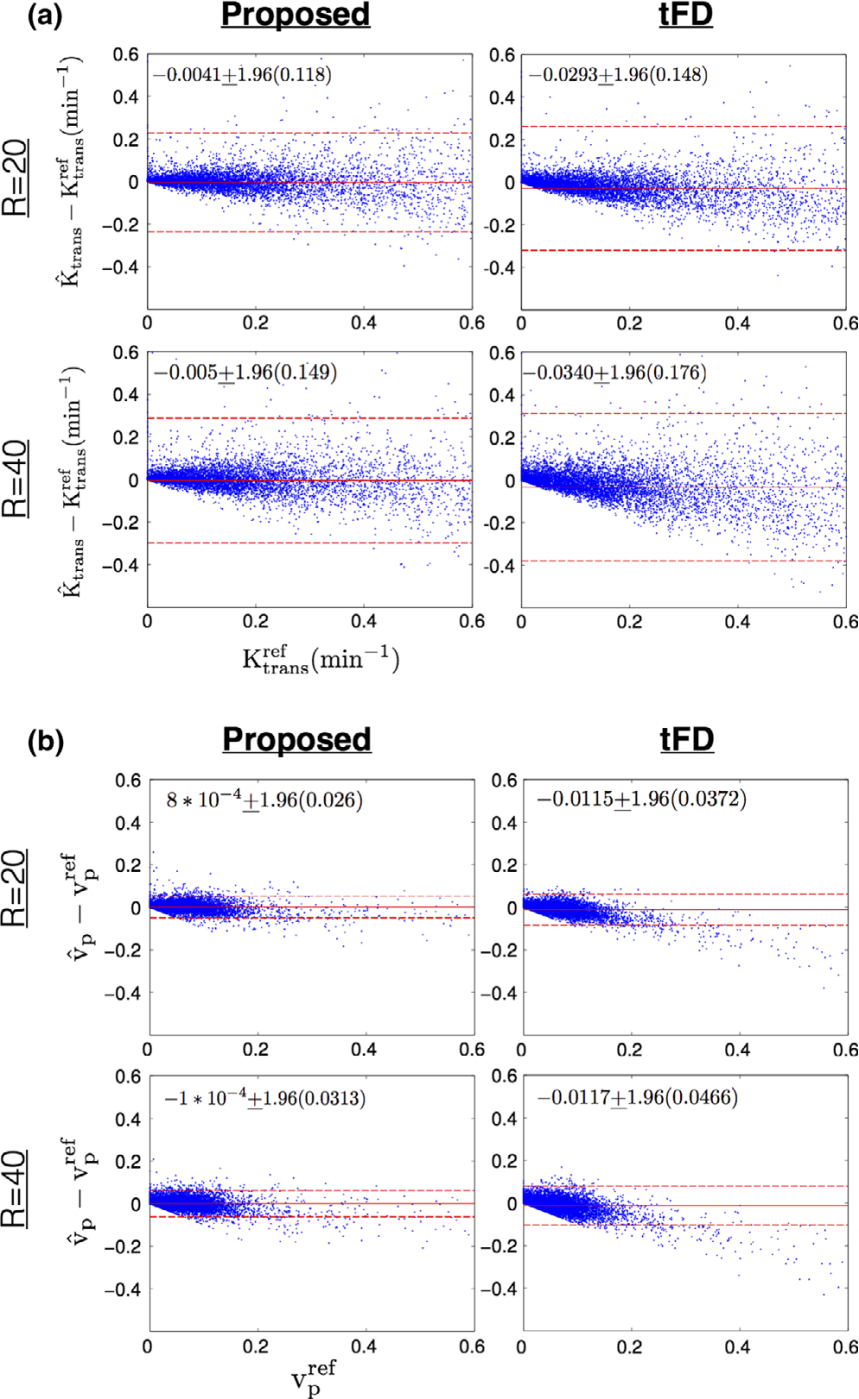
Bland‐Altman plots of (a) the difference between estimated K^trans^ (at R = 20, R = 40) and reference K^trans^; (b) the difference between estimated vp (at R = 20, R = 40) and reference vp; for the proposed (left column) and tFD (right column) reconstructions. Each dot corresponds to one pixel within the tumor ROIs of all the 12 cases. The mean and 1.96 times the standard deviation (μ ± 1.96σ) of the difference entities are quantitatively shown. These are also qualitatively marked by the solid red and dotted red lines. As seen from the plots, the proposed approach had lower bias (μ) and uncertainty (σ) in estimating K^trans^, and v_p_ in comparison to tFD. tFD depicted a systematic bias in underestimating K^trans^, and v_p_ in comparison to the proposed approach This can also be noted from the qualitative comparisons in Fig. 9. [Color figure can be viewed at http://www.wileyonlinelibrary.com]

Figure 10 shows Bland‐Altman plots of the difference between estimated TK parameters (at R = 20, R = 40) and reference TK parameters on all the 12 cases combined. In comparison to tFD, the proposed approach showed reduced bias and reduced certainty during estimation of K^trans^, and v_p_. A systematic bias of under estimating K^trans^, and v_p_ was present in tFD, which is also qualitatively shown in Fig. 8.

**Figure 11:**
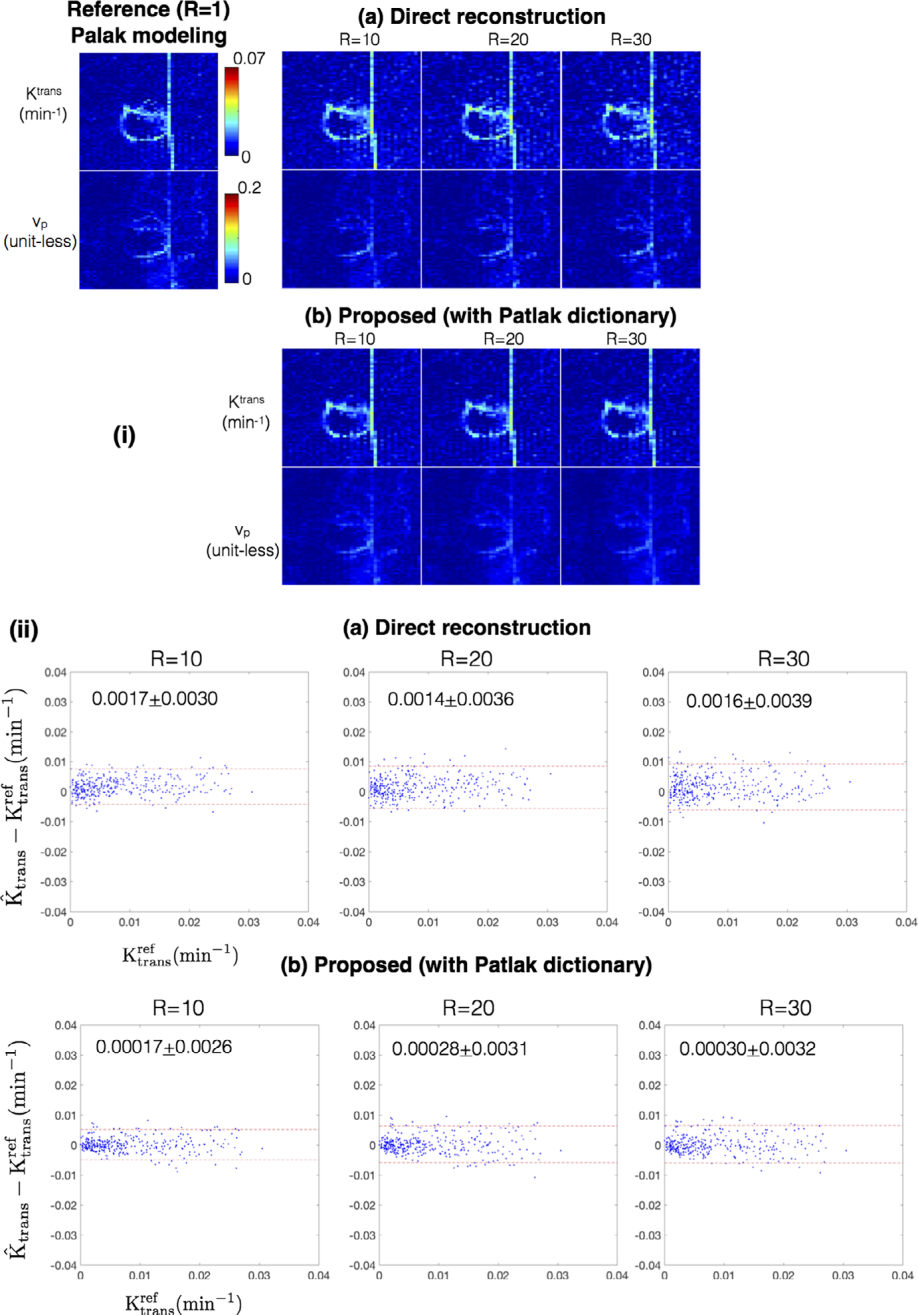
(i) Comparison of K^trans^ and v_p_ derived from the methods of direct reconstruction of Patlak parameters 36, and the proposed approach with a Patlak dictionary at R = 10, 20, 30 against the reference fully sampled reconstruction followed by Patlak modeling (R = 1). (ii) shows the Bland‐Altman plots of the difference between estimated K^trans^ (at R = 10, 20, 30) and the reference K^trans^ for the direct reconstruction and proposed reconstruction with the Patlak dictionary. Each dot corresponds to one pixel within the tumor ROI of case 7 as marked in Fig. 2. The mean and 1.96 times the standard deviation (μ ± 1.96σ) of the difference entities are quantitatively shown in (ii). Both direct reconstruction, and the proposed dictionary reconstruction provides good spatial fidelity in the reconstructed maps (in i). From the Bland‐Altman plots in (ii), the proposed approach demonstrated subtle improvements over the direct reconstruction in terms of a smaller bias and reduced noise amplification. [Color figure can be viewed at http://www.wileyonlinelibrary.com]

Figure 11 shows the comparison of the estimated TK parameters from the proposed approach with Patlak dictionary against the direct Patlak parameter estimation at R = 20, 30, 40 on case 7. Both the proposed approach and the direct reconstruction provided good spatial fidelity of the TK maps as depicted in Fig. 11(i). The proposed approach however demonstrated subtle benefits in terms of reduced bias, and reduced noise amplification as shown in the Bland‐Altman plots of Fig. 11(ii).

## 4 Discussion

We have developed a new DCE‐MRI reconstruction approach that applies kinetic models routinely used in post‐processing as temporal constraints during reconstruction. Based on simulation studies, we deduced a relation between sparsity parameter q in k‐SVD to the complexity of the kinetic model. We have demonstrated equivalence of Patlak and ETK models with dictionaries constructed respectively with q = 2 and q = 3. This approach exploits the smooth time intensity DCE patterns by using temporal basis functions derived from a kinetic model. This is in contrast to generic off‐the‐shelf transform bases that are blind to the kinetic model behavior of the time intensity profiles. We also proposed a robust multiscale iterative optimization algorithm to solve the resulting l0 norm based nonconvex objective function. We empirically demonstrated robustness to local minima. The tFD approach has a convex formulation with a guaranteed global minimum, and therefore, no multiscale optimization heuristics were applied during tFD minimization. *In‐vivo* validation with 12 brain tumor cases demonstrated superior recovery performance with the proposed method compared to tFD (reduced bias, uncertainty in kinetic mapping, and better spatial fidelity of kinetic maps) at up to R = 40.

The proposed framework can be extended in several ways. A uniform grid of kinetic parameters was used in this study to generate the library of possible concentration profiles from a chosen kinetic model. However, it is possible to perform application‐specific discretization of the kinetic parameters to improve sensitivity and accuracy in modeling time curves that lie in a particular zone in the kinetic parameter space. We have demonstrated that for the 2‐parameter Patlak model, a choice of two temporal bases from the dictionary was adequate to reliably model the concentration temporal profiles. For the 3‐parameter ETK model, three temporal bases were adequate. We expect that if this approach is extended to more complex models (e.g., fast exchange, shutter speed, two compartment exchange model), the complexity of the bases representation may also increase. Increasing model complexity is expected to place more stringent limits on the maximum achievable acceleration rates.

This study demonstrates the utility of the kinetic model‐based reconstruction approach in the application of brain tumor imaging. The framework may be extended to DCE‐MRI of other diseases and body parts by appropriately considering different ranges of kinetic parameters and kinetic models. Other applications would also warrant validation with digital reference objects specific to that body part, disease, and kinetic parameter ranges. Complementary constraints such as spatial sparsity could be added to further improve the recovery.

### 4.1 Limitations of the study

A limitation of this feasibility study is the use of population‐averaged AIF.^43^ Population‐averaged AIFs are known to produce a potential bias in the final kinetic maps,^57^ however in this study, the bias identically affects the reference maps, and maps produced by the proposed reconstruction and the temporal finite difference reconstruction that is used for comparison. The framework can be extended to account for patient‐specific AIFs. For instance, the full patient‐specific AIFs can be obtained from a preprocessing reconstruction or could be characterized by including richer dictionaries that parameterize the shape and amplitude of the AIF.^58^, ^59^ Our preliminary findings show that with whole brain scans and transform sparsity reconstruction, we could extract good fidelity AIFs that are free of inflow enhancement artifacts.^54^ Such a reconstruction could potentially be a preliminary pre‐processing step. These extensions are a scope of our future work.

In this study, our DCE‐MRI protocol employed a flip angle of 15°, and *in‐vivo* data were acquired at ½ dose (0.05 mMol/kg). With these settings, there exists some nonlinearity in mapping between concentration profiles and signal intensity time profiles at concentrations > 1.0 mM. The linearity can however be improved with the use of flip angles ≥ 25°. R2* effects were not included in the forward imaging model.^60^ Our DCE‐MRI scans were performed with ½ dose (0.05 mmol/kg), and used a short TE of 2 ms. We have examined several clinical datasets at our institution and have found phase and R2* effects to be insignificant in tissue and in vessels. We therefore did not consider R2* or off‐resonance effects, but these could be easily added to the forward model.

v_e_ has shown clinical value in studies with long scan times of 10 minutes or more (e.g., Ref. [61]). At our institution (and most of our peer institutions) the standard of care brain tumor protocol utilizes a DCE‐MRI scan time of 5 min, which is insufficient to recover v_e_. This is documented in the literature where estimation of v_e_ had high uncertainty with short scan times,^56^ and longer scan times o upto 10 min are recommended.^56^, ^62^ We have included a range of values for v_e_ in the library because accounting for backflux is known to improve the estimation of K^trans^ and v_p_.^41^

In this study, we have only focused our analysis only on tumor ROIs. We intend to perform comprehensive statistical studies against controls (nonleaking areas from the whole brain) in a future study with prospective under‐sampling that can enable whole brain coverage.

Comparison of the proposed approach against direct reconstruction of Patlak parameters showed subtle improvements in estimating the TK parameters with less noise amplification, and reduced bias on a single dataset. Comprehensive evaluation against the direct reconstruction method when using nonlinear models and multiple datasets were not performed in this study as this warrants establishing a detailed study of optimization routines in direct reconstruction (e.g., Newton based,^36^ variable splitting^37^). An important distinction of the proposed work is that it decouples computationally expensive kinetic parameter estimation from the reconstruction of concentration vs time profiles. Kinetic parameter estimation need only be performed once as a final step. In contrast, direct reconstruction, as described in Guo et al.,^37^ requires kinetic parameter estimation at every iteration. This itself can require on the order of minutes to hours for typical DCE‐MRI datasets with sizes of at least 128 × 128 pixels and 50 time frames. Precise reconstruction times depend on the choice of optimization solver and their implementation (e.g., efficiency and whether they exploit GPUs). We have not performed detailed comparisons of reconstruction times, as this is beyond the scope of this feasibility study.

The T1 maps were estimated prior to reconstruction using DESPOT1 with three flip angles.^63^ However, using fully sampled data, the joint estimation of T1 and kinetic parameter maps has recently been shown to improve accuracy of DCE kinetic parameter maps.^64^ An extension of the proposed framework to include joint T1 estimation would warrant the inclusion of multiple T1‐based simulated concentration curves in the library, and exploration of superior learning approaches (alternate to k‐SVD) that offer better compression capabilities to efficiently represent a richer library. Furthermore, there could be a number of approaches to improve pre‐contrast T1 mapping in a separate step. For instance, increasing the number of flip angle measurements, use of constrained imaging methods (e.g., model‐based reconstruction, MR fingerprinting).

The proposed approach has similarities and important distinctions with prior art. Similar to MR‐fingerprinting,^48^, ^65^ our approach exploits physical models for reconstruction. However, it does not modify the acquisition parameter settings. It takes a two‐step approach of first reconstructing the concentration time profiles, and then estimating the kinetic parameters in a final step. In comparison to MR‐Fingerprinting, our approach is sensitive to motion because the basis functions do not account for motion. However, if reasonable estimates of the motion deformation fields are known or can be estimated from the data, it can be corrected by integration into the forward model.^66^, ^67^

Our experiments in this work show incoherence along time benefits the reconstruction. However, a detailed evaluation of various sampling pattern choices including coherent sampling, and evaluation against incoherent sampling is yet to be done, and is a scope of future work.

Data inconsistencies such as motion, or B1 non‐uniformity, may violate the assumption of the appropriateness of the kinetic model on the concentration time profiles. This is equally true with existing compressed sensing methods. However, the framework can seamlessly accommodate prior information in the forward model to improve data consistency (e.g., integration of motion maps, B1 maps). The proposed reconstruction assumes the chosen kinetic model to be appropriate to the data. While the kinetic model of choice can be motivated based on the application at hand, the framework is flexible to generate comprehensive libraries from more than one kinetic model. Future application‐specific studies with chosen kinetic models are however needed to deduce the relation between the complexity of the library and the sparsity parameter (q) during dictionary generation, and subsequently acceleration capabilities.

## Conflict of interest

The authors have no conflict of interest to disclose.

## References

[1] Heye AK , Culling RD , Valdés Hernández MdC , Thrippleton MJ , Wardlaw JM . Assessment of blood–brain barrier disruption using dynamic contrast‐enhanced MRI. A systematic review. NeuroImage. 2014;6:262–274. 25379439 10.1016/j.nicl.2014.09.002 PMC4215461

[2] Roberts HC , Roberts TP , Brasch RC , Dillon WP . Quantitative measurement of microvascular permeability in human brain tumors achieved using dynamic contrast‐enhanced MR imaging: correlation with histologic grade. AJNR Am J Neuroradiol. 2000;21:891–899. 10815665 PMC7976746

[3] Jain R . Measurements of tumor vascular leakiness using DCE in brain tumors: clinical applications. NMR Biomed. 2013;26:1042–1049. 23832526 10.1002/nbm.2994

[4] Gaitan MI , Shea CD , Evangelou IE , et al. Evolution of the blood‐brain barrier in newly forming multiple sclerosis lesions. Ann Neurol. 2011;70:22–29. 21710622 10.1002/ana.22472 PMC3143223

[5] Cramer SP , Larsson HBW . Accurate determination of blood‐brain barrier permeability using dynamic contrast‐enhanced T1‐weighted MRI: a simulation and in vivo study on healthy subjects and multiple sclerosis patients. J Cereb blood flow Metab. 2014:34:1655–1665. 25074746 10.1038/jcbfm.2014.126 PMC4269724

[6] Starr JM , Wardlaw J , Ferguson K , et al.Increased blood‐brain barrier permeability in type II diabetes demonstrated by gadolinium magnetic resonance imaging. J Neurol Neurosurg Psychiatry. 2003;74:70–76. 12486269 10.1136/jnnp.74.1.70 PMC1738177

[7] Montagne A , Barnes SR , Sweeney MD , et al. Blood‐Brain barrier breakdown in the aging human hippocampus. Neuron. 2015;85:296–302. 25611508 10.1016/j.neuron.2014.12.032 PMC4350773

[8] Ah‐See M‐LW , Makris A , Taylor NJ , et al. Early changes in functional dynamic magnetic resonance imaging predict for pathologic response to neoadjuvant chemotherapy in primary breast cancer. Clin Cancer Res. 2008;14:6580–6589. 18927299 10.1158/1078-0432.CCR-07-4310

[9] Li X , Arlinghaus LR , Ayers GD , et al. DCE‐MRI analysis methods for predicting the response of breast cancer to neoadjuvant chemotherapy: pilot study findings. Magn Reson Med. 2013;71:1592–1602. 23661583 10.1002/mrm.24782 PMC3742614

[10] Verma S , Turkbey B , Muradyan N , et al. Overview of dynamic contrast‐enhanced MRI in prostate cancer diagnosis and management. Am J Roentgenol. 2012;198:1277–1288. 22623539 10.2214/AJR.12.8510 PMC6309691

[11] Bin CB , Shih TTF . DCE‐MRI in hepatocellular carcinoma‐clinical and therapeutic image biomarker. World J Gastroenterol. 2014;20:3125–3134. 24695624 10.3748/wjg.v20.i12.3125 PMC3964384

[12] D’Arcy JA , Collins DJ , Rowland IJ , Padhani AR , Leach MO . Applications of sliding window reconstruction with cartesian sampling for dynamic contrast enhanced MRI. NMR Biomed. 2002;15:174–183. 11870913 10.1002/nbm.755

[13] Le Y , Kroeker R , Kipfer HD , Lin C . Development and evaluation of TWIST Dixon for dynamic contrast‐enhanced (DCE) MRI with improved acquisition efficiency and fat suppression. J Magn Reson Imaging. 2012;36:483–491. 22544731 10.1002/jmri.23663

[14] Saranathan M , Rettmann DW , Hargreaves BA , Clarke SE , Vasanawala SS . DIfferential subsampling with cartesian ordering (DISCO): a high spatio‐temporal resolution dixon imaging sequence for multiphasic contrast enhanced abdominal imaging. J Magn Reson Imaging. 2012;35:1484–1492. 22334505 10.1002/jmri.23602 PMC3354015

[15] Wieben O , Velikina J , Block WF , et al. Highly constrained back projection (HYPR): theory and potential MRI applications. Med Phys. 2006;14:2006–2006.

[16] Han S , Paulsen JL , Zhu G , et al. Temporal/spatial resolution improvement of in vivo DCE‐MRI with compressed sensing‐optimized FLASH. Magn Reson Imaging. 2012;30:741–752. 22465192 10.1016/j.mri.2012.02.001 PMC3792168

[17] Lebel RM , Jones J , Ferre J‐C , Law M , Nayak KS . Highly accelerated dynamic contrast enhanced imaging. Magn Reson Med. 2014;71:635–644. 23504992 10.1002/mrm.24710

[18] Guo Y , Lebel RM , Zhu Y , et al. High‐resolution whole‐brain DCE‐MRI using constrained reconstruction: prospective clinical evaluation in brain tumor patients. Med Phys. 2016;43:2013–2023. 27147313 10.1118/1.4944736 PMC4826379

[19] Feng L , Grimm R , Block KT , et al. Golden‐angle radial sparse parallel MRI: combination of compressed sensing, parallel imaging, and golden‐angle radial sampling for fast and flexible dynamic volumetric MRI. Magn Reson Med. 2013;72:707–717. 24142845 10.1002/mrm.24980 PMC3991777

[20] Rosenkrantz AB , Geppert C , Grimm R , et al. Dynamic contrast‐enhanced MRI of the prostate with high spatiotemporal resolution using compressed sensing, parallel imaging, and continuous golden‐angle radial sampling: preliminary experience. J Magn Reson Imaging. 2015;41:1365–1373. 24833417 10.1002/jmri.24661 PMC4233205

[21] Zhang T , Cheng JY , Potnick AG , et al. Fast pediatric 3D free‐breathing abdominal dynamic contrast enhanced MRI with high spatiotemporal resolution. J Magn Reson Imaging. 2015;41:460–473. 24375859 10.1002/jmri.24551 PMC4065644

[22] Lingala SG , Jacob M . Blind compressive sensing dynamic MRI. IEEE Trans Med Imaging. 2013;32:1132–1145. 23542951 10.1109/TMI.2013.2255133 PMC3902976

[23] Awate SP , Dibella EVR . Spatiotemporal dictionary learning for undersampled dynamic MRI reconstruction via joint frame‐based and dictionary‐based sparsity. In: Proceedings ‐ International Symposium on Biomedical Imaging; 2012:318‐321. 10.1109/ISBI.2012.6235548

[24] Wang Y , Ying L . Compressed sensing dynamic cardiac cine MRI using learned spatiotemporal dictionary. IEEE Trans Biomed Eng. 2014;61:1109–1120. 24658236 10.1109/TBME.2013.2294939

[25] Caballero J , Price AN , Rueckert D , Hajnal JV . Dictionary learning and time sparsity for dynamic MR data reconstruction. IEEE Trans Med Imaging. 2014;33:979–994. 24710166 10.1109/TMI.2014.2301271

[26] Wang G , Qi J . Direct estimation of kinetic parametric images for dynamic PET. Theranostics. 2013;3:802–815. 24396500 10.7150/thno.5130 PMC3879057

[27] Kamasak ME , Bouman CA , Morris ED , Sauer K . Direct reconstruction of kinetic parameter images from dynamic PET data. IEEE Trans Med Imaging. 2005;24:636–650. 15889551 10.1109/TMI.2005.845317

[28] Rahmim A , Tang J , Zaidi H . Four‐dimensional (4D) image reconstruction strategies in dynamic PET: beyond conventional independent frame reconstruction. Med Phys. 2009;36:3654–3670. 19746799 10.1118/1.3160108

[29] Li T , Thorndyke B , Schreibmann E , Yang Y , Xing L . Model‐based image reconstruction for four‐dimensional PET. Med Phys. 2006;33:1288–1298. 16752564 10.1118/1.2192581

[30] Doneva M , Börnert P , Eggers H , Stehning C , Sénégas J , Mertins A . Compressed sensing reconstruction for magnetic resonance parameter mapping. Magn Reson Med. 2010;64:1114–1120. 20564599 10.1002/mrm.22483

[31] Sumpf TJ , Uecker M , Boretius S , Frahm J . Model‐based nonlinear inverse reconstruction for T2 mapping using highly undersampled spin‐echo MRI. J Magn Reson Imaging. 2011;34:420–428. 21780234 10.1002/jmri.22634

[32] Huang C , Graff CG , Clarkson EW , Bilgin A , Altbach MI . T2 mapping from highly undersampled data by reconstruction of principal component coefficient maps using compressed sensing. Magn Reson Med. 2012;67:1355–1366. 22190358 10.1002/mrm.23128 PMC3311721

[33] Ben‐Eliezer N , Sodickson DK , Block KT . Rapid and accurate T2 mapping from multi‐spin‐echo data using bloch‐simulation‐based reconstruction. Magn Reson Med. 2015;73:809–817. 24648387 10.1002/mrm.25156 PMC4169365

[34] Awate SP , DiBella EVR , Tasdizen T , Whitaker RT . Model‐based image reconstruction for dynamic cardiac perfusion MRI from sparse data. In: Annual International Conference of the IEEE Engineering in Medicine and Biology ‐ Proceedings; 2006:936‐941. 10.1109/IEMBS.2006.260363 17946012

[35] Zhao L , Fielden SW , Feng X , Wintermark M , Mugler JP , Meyer CH . Rapid 3D dynamic arterial spin labeling with a sparse model‐based image reconstruction. NeuroImage. 2015;121:205–216. 26169322 10.1016/j.neuroimage.2015.07.018 PMC4615585

[36] Guo Y , Lingala SG , Zhu Y , Lebel RM , Nayak KS . Direct estimation of tracer‐kinetic parameter maps from highly undersampled brain dynamic contrast enhanced MRI. Magn Reson Med. 2017;78:1566–1578. 27859563 10.1002/mrm.26540 PMC5435562

[37] Guo Y , Lingala SG , Bliesener Y , Lebel RM , Zhu Y , Nayak KS . Joint arterial input function and tracer kinetic parameter estimation from undersampled dynamic contrast‐enhanced MRI using a model consistency constraint. Magn Reson Med. 2018;79:2804–2815. 28905411 10.1002/mrm.26904 PMC5821580

[38] Welsh CL , Dibella EVR , Adluru G , Hsu EW . Model‐based reconstruction of undersampled diffusion tensor k‐space data. Magn Reson Med. 2013;70:429–440. 23023738 10.1002/mrm.24486 PMC4469271

[39] Bilgic B , Chatnuntawech I , Setsompop K , et al. Fast dictionary‐based reconstruction for diffusion spectrum imaging. IEEE Trans Med Imaging. 2013;32:2022–2033. 23846466 10.1109/TMI.2013.2271707 PMC4689148

[40] Aharon M , Elad M , Bruckstein A . k‐SVD: an algorithm for designing overcomplete dictionaries for sparse representation. IEEE Trans Signal Process. 2006;54:4311–4322.

[41] Sourbron SP , Buckley DL . On the scope and interpretation of the Tofts models for DCE‐MRI. Magn Reson Med. 2011;66:735–745. 21384424 10.1002/mrm.22861

[42] Patlak CS , Blasberg RG . Graphical evaluation of blood to brain barrier transfer constants from multiple time uptake data. Generalizations. J Cereb Blood Flow Metab. 1985;5:584–590. 4055928 10.1038/jcbfm.1985.87

[43] Parker GJM , Roberts C , Macdonald A , et al. Experimentally‐derived functional form for a population‐averaged high‐temporal‐resolution arterial input function for dynamic contrast‐enhanced MRI. Magn Reson Med. 2006;56:993–1000. 17036301 10.1002/mrm.21066

[44] Li KL , Zhu XP , Waterton J , Jackson A . Improved 3D quantitative mapping of blood volume and endothelial permeability in brain tumors. J Magn Reson Imaging. 2000;12:347–357. 10931600 10.1002/1522-2586(200008)12:2<347::aid-jmri19>3.0.co;2-7

[45] Pati YCC , Rezaiifar R , Krishnaprasad PSS .Orthogonal matching pursuit: recursive function approximation with applications to wavelet decomposition. *Proc 27th Asilomar Conf. Signals, Syst Comput* 1993;1–5. 10.1109/ACSSC.1993.342465

[46] Sotiras A , Davatzikos C , Paragios N . Deformable medical image registration: a survey. IEEE Trans Med Imaging. 2013;32:1153–1190. 23739795 10.1109/TMI.2013.2265603 PMC3745275

[47] Pierre EY , Ma D , Chen Y , Badve C , Griswold MA . Multiscale reconstruction for MR fingerprinting. Magn Reson Med. 2015;75:2481–2492. 26132462 10.1002/mrm.25776 PMC4696924

[48] Hamilton JI , Jiang Y , Chen Y , et al. MR fingerprinting for rapid quantification of myocardial T1, T2, and proton spin density. Magn Reson Med. 2016;7:1446–1458. 10.1002/mrm.26216 PMC5045735 27038043

[49] Barnes SR , Ng TSC , Santa‐Maria N , Montagne A , Zlokovic BV , Jacobs RE . ROCKETSHIP: a flexible and modular software tool for the planning, processing and analysis of dynamic MRI studies. BMC Med Imaging. 2015;15:19. 26076957 10.1186/s12880-015-0062-3 PMC4466867

[50] Bosca R , Jackson E . Creating an anthropomorphic digital MR phantom—an extensible tool for comparing and evaluating quantitative imaging algorithms. Phys Med Biol. 2016;61:2016. 10.1088/0031-9155/61/2/974 26738776

[51] Zhu Y , Guo Y , Lingala SG , Marc Lebel R , Law M , Nayak KS . GOCART: GOlden‐angle CArtesian randomized time‐resolved 3D MRI. Magn Reson Imaging. 2015;34:940–950. 26707849 10.1016/j.mri.2015.12.030

[52] Wen PY , Macdonald DR , Reardon DA , et al. Updated response assessment criteria for high‐grade gliomas: response assessment in neuro‐oncology working group. J Clin Oncol. 2010;28:1963–1972. 20231676 10.1200/JCO.2009.26.3541

[53] Bagher‐Ebadian H , Jain R , Nejad‐Davarani SP , et al. Model selection for DCE‐T1 studies in glioblastoma. Magn Reson Med. 2012;68:241–251. 22127934 10.1002/mrm.23211 PMC3292667

[54] Lebel R , Nallapareddy N , Lingala SG , Frayne R , Nayak KS . Automatic bolus detection for dynamic contrast enhaced imaging with sparse sampling. In: Proceedings of the MR Angiography Club; 2016:76.

[55] Daniel BL , Yen YF , Glover GH , et al. Breast disease: dynamic spiral MR imaging. Popul (English Ed). 1998;209:499–509. 10.1148/radiology.209.2.9807580 9807580

[56] Artzi M , Liberman G , Nadav G , et al. Optimization of DCE‐MRI protocol for the assessment of patients with brain tumors. Magn Reson Imaging. 2016;34:1242–1247. 27451404 10.1016/j.mri.2016.07.003

[57] Port RE , Knopp MV , Brix G . Dynamic contrast‐enhanced MRI using Gd‐DTPA: interindividual variability of the arterial input function and consequences for the assessment of kinetics in tumors. Magn Reson Med. 2001;45:1030–1038. 11378881 10.1002/mrm.1137

[58] Calamante F , Gadian DG . Connelly a. Delay and dispersion effects in dynamic susceptibility contrast MRI: simulations using singular value decomposition. Magn Reson Med. 2000;44:466–473. 10975900 10.1002/1522-2594(200009)44:3<466::aid-mrm18>3.0.co;2-m

[59] Willats L , Connelly A , Christensen S , Donnan GA , Davis SM , Calamante F . The role of bolus delay and dispersion in predictor models for stroke. Stroke. 2012;43:1025–1031. 22343645 10.1161/STROKEAHA.111.635888

[60] Schabel MC , Parker DL . Uncertainty and bias in contrast concentration measurements using spoiled gradient echo pulse sequences. Phys Med Biol. 2008;53:23–45. 18421121 10.1088/0031-9155/53/9/010 PMC2894639

[61] Li X , Zhu Y , Kang H , et al. Glioma grading by microvascular permeability parameters derived from dynamic contrast‐enhanced MRI and intratumoral susceptibility signal on susceptibility weighted imaging Head & neck imaging. Cancer Imaging. 2015;15:4. 25889239 10.1186/s40644-015-0039-z PMC4389664

[62] Tofts PS , Parker GJM . DCE‐MRI: acquisition and analysis techniques In: Barker PB, Golay X, Zaharchuk G, eds. Clinical Perfusion MRI: Techniques and Applications. Cambridge: Cambridge University Press; 2013:58–74. 10.1017/CBO9781139004053.006.

[63] Deoni SCL , Peters TM , Rutt BK . High‐resolution T1 and T2 mapping of the brain in a clinically acceptable time with DESPOT1 and DESPOT2. Magn Reson Med. 2005;53:237–241. 15690526 10.1002/mrm.20314

[64] Dickie B , Banerji A , Kershaw L , et al. Improved accuracy and precision of tracer kinetic parameters by joint fitting to variable flip angle and dynamic contrast enhanced MRI data. Magn Reson Med. 2016;76:1270–1281. 26480291 10.1002/mrm.26013

[65] Ma D , Gulani V , Seiberlich N , et al. Magnetic resonance fingerprinting. Nature. 2013;495:187–192. 23486058 10.1038/nature11971 PMC3602925

[66] Lingala SG , DiBella E , Jacob M . Deformation corrected compressed sensing (DC‐CS): a novel framework for accelerated dynamic MRI. IEEE Trans Med Imaging. 2014;34:72–85. 25095251 10.1109/TMI.2014.2343953 PMC4411243

[67] Chen X , Salerno M , Yang Y , Epstein FH . Motion‐compensated compressed sensing for dynamic contrast‐enhanced MRI using regional spatiotemporal sparsity and region tracking: Block low‐rank sparsity with motion‐guidance (BLOSM). Magn Reson Med. 2014;72:1028–1038. 24243528 10.1002/mrm.25018 PMC4097987

